# The efficacy of Tai Chi for intervention treatment of myocardial infarction: A protocol for systematic review and meta-analysis

**DOI:** 10.1097/MD.0000000000027584

**Published:** 2021-11-19

**Authors:** Shanshan Wu, Zuosheng Lu, Zhaowei Li, Yuezhong Li

**Affiliations:** a School of Physical Education & Sports Science, South China Normal University, Guangdong, China; b College of Physical Education and Health, Guangzhou University of Chinese Medicine, Guangdong, China.

**Keywords:** cardiac rehabilitation, meta-analysis, myocardial infarction, prognosis, Tai Chi

## Abstract

**Background:**

This protocol for systematic review and meta-analysis aims at assessing the clinical evidence regarding the efficacy of Tai Chi interventions in patients with myocardial infarction (MI).

**Methods:**

Literature retrieval will use the Cochrane Library, Web of Science, PubMed, Embase, Allied and Complementary Medicine Database, China Biomedical Literature Database, China National Knowledge Infrastructure, China Science and Technology Journal Database, Wanfang Database, and Ongoing Clinical Trials Database. Our search strategy was based on a string of text words, Medical Subject Headings, and subject headings indicative of Tai Chi. The search strings included: tai chi chuan, taiji, and taiji quan interventions in myocardial infarction; MI infarcts; myocardial infarcts; myocardial; coronary artery disease; coronary arteriosclerosis; acute coronary syndromes; and coronary syndromes. Quality assessment of the included studies was evaluated using the Cochrane risk of bias assessment tool. Statistical analyses were performed using RevMan 5.4 software.

**Results:**

The findings of this study will be submitted to peer-reviewed journals for publication.

**Conclusion:**

This study will provide reliable evidence regarding the efficacy of Tai Chi in patients with MI and provide up-to-date evidence for its application.

## 1 Introduction

Cardiovascular disease poses tremendous burden on public health, as well as the global economy.^[1,2]^ According to the World Health Organization, an estimated 17.5 million people died from cardiovascular disease in 2012, accounting for 31% mortality globally with an annual cost of $193.1 billion in health-care management and about $123 billion in productivity loss as a result of premature death.^[3]^ Myocardial infarction (MI) is a leading cause of morbidity and mortality worldwide with known complications including congestive heart failure, functional and structural myocardial abnormalities, reinfarction, and death.^[4,5]^

Although challenges remain in preventing and treating MI, regular exercise is recognized as a primary strategy for preventing a heart attack. However, cardiac events may also be triggered during extreme physical activities, such as high-intensity exercise or competitive sport. Therefore, the role played by the incorporation of exercise in health care regimens should be thoroughly investigated. Also, ensuring proper warm-up and cool-down procedures, as well as adequate hydration during exercise, is crucial. These procedures are highly recommended by both the American Heart Association and the European Association of Preventive Cardiology. Tai Chi, one of the most widely practiced mind-body exercises, is reportedly a beneficial way to prevent MI and promote recovery after MI.^[6]^

In the past few decades, the beneficial effects of Tai Chi on health have been highly reported. As a result, several clinical studies have been performed to assess the efficacy of Tai Chi on a wide range of disease conditions, including diabetes, lower limb proprioception, Parkinson disease, and stroke.^[7–9]^ Tai Chi is also beneficial for patients with heart disease. The effects of Tai Chi are not limited to cardiac rehabilitation; it also aids in heart rate variability and stress management.^[10]^ This systematic review aims at assessing the clinical evidence regarding the efficacy of Tai Chi interventions in patients with MI. Notably, the meta-analysis data search was conducted in both English and Chinese databases, and therefore, includes the most up-to-date research and information on the findings of recent related studies.

## 2 Methods

The presentation of methods and results in this systematic review was performed according to the evaluation guidelines for health care interventions provided in the Preferred Reporting Items for Systematic Reviews and Meta-Analyses Protocol.^[11]^ It was registered in OSF (OSF registration number: 10.17605/OSF.IO/C52JD). Ethical approval is not required because it is on the basis of published studies.

### 2.1 Search strategy

Literature retrieval will use the Cochrane Library, Web of Science, PubMed, Embase, Allied and Complementary Medicine Database, China Biomedical Literature Database, China National Knowledge Infrastructure, China Science and Technology Journal Database, Wanfang Database, and Ongoing Clinical Trials Database. We consider articles published between database initiation and October 2021. There were no restrictions on languages. Our search strategy was based on a string of text words, Medical Subject Headings, and subject headings indicative of Tai Chi. The search strings included: tai chi chuan, taiji, and taiji quan interventions in myocardial infarction; MI infarcts; myocardial infarcts; myocardial; coronary artery disease; coronary arteriosclerosis; acute coronary syndromes; and coronary syndromes.

### 2.2 Inclusion criteria

Only randomized controlled trials that met the following inclusion criteria were used: published in English or Chinese; included patients with MI, both ST-segment elevation MI and non-ST-segment elevation MI, regardless of whether the patients were undergoing percutaneous coronary intervention; and compared an intervention group that performed Tai Chi alone or with other interventions with a control group that performed other exercises that received usual care or that did not use any intervention. We included studies published in peer-review journals, dissertations indexed in bibliographical databases, and unpublished full manuscripts. We also included studies investigating the 6-minute walk test and left ventricular ejection fraction as the 2 primary outcomes; and N-terminal pro B type natriuretic peptide level, pro-B type natriuretic peptide level, Medical Outcomes Study short-form health survey, quality of life, activities of daily living scale, and sense of coherence scale as the secondary outcomes. Studies were excluded if: they were not fully accessible, they were duplicated citations, and they possessed a poor quality score as per the stated criteria.

### 2.3 Data extraction and classification

The following data were extracted for each article: bibliographical data, including authors and year of publication; clinical trial features such as sample size, study flow, recruitment method, criteria for inclusion and exclusion, type of control group, primary measures and functioning outcomes, time and point of assessments, and duration of the intervention; participant characteristics such as age, sex, and so on; patient background, including country and type of medical center; nature of human guidance (e.g., personal guidance, discussion group, unguided); form of guidance provider (e.g., therapist-led, peer-led, unguided); and study drop-out rate and handling of missing data.

### 2.4 Risk of bias assessment

Two investigators will separately assess the risk of bias of the included studies using the Cochrane risk of bias assessment tool. The evaluation of each study mainly included the following 7 aspects: random sequence generation, allocation hiding, blinding of participants and personnel, blinding of outcome assessment, incomplete outcome data, selective outcome reporting, and other biases. Finally, the bias of the study will be rated on 3 levels: “low”, “high”, and “ambiguous”. These even domains will be separately appraised by 2 reviews, and discrepancies will be addressed by consulting a third reviewer.

### 2.5 Statistical analysis

In this study, we will apply RevMan 5.4 software (Microsoft, USA) for statistical analysis. The risk ratio and 95% confidence intervals were collected for enumeration data, while the mean difference or standardized mean difference and 95% confidence intervals were used to calculate continuous outcome data. Heterogeneity among trials will be identified by the I^2^ and chi-squared test statistics. If the included studies have high heterogeneity (I^2^ > 50%), we will use a random-effects model for pooling data across studies. Otherwise, a fixed-effects model will be used. To evaluate publication bias, we will construct a funnel plot using Cochrane software (Microsoft, USA) if the number of included studies is sufficient (>10 studies). A symmetrical funnel plot indicates no possibility of publication bias, whereas an asymmetrical funnel plot indicates a high possibility of publication bias. If we identify publication bias through analysis of the funnel plot, we will discuss possible reasons such as small-study effects.

## 3 Discussion

Cardiac rehabilitation has been a successful approach to improve the functional capacity, recovery, and psychological well-being in patients after MI.^[12–14]^ However, only a small number of patients have access to rehabilitation programs. In this setting, Tai Chi can be an appealing alternative for cardiac rehabilitation. Different from the traditional form of cardiac rehabilitation, Tai Chi exercise requires patients to focus on their breath and body movement instead of trying their best to achieve predetermined goals such as heart rate or exercise intensity.^[15,16]^ This study will provide reliable evidence regarding the efficacy of Tai Chi in patients with MI and provide up-to-date evidence for its application.

## Author contributions

**Conceptualization:** Zuosheng Lu.

**Data curation:** Zuosheng Lu.

**Funding acquisition:** Yuezhong Li.

**Investigation:** Zhaowei Li.

**Methodology:** Shanshan Wu.

**Writing – original draft:** Shanshan Wu.

**Writing – review & editing:** Yuezhong Li.
